# Single target formulation of thin film 58S bioactive glasses *via* magnetron sputtering with custom targets from spray-dried bioactive glass powders

**DOI:** 10.1039/d5ra02728d

**Published:** 2025-06-09

**Authors:** Yao-Chong Fan, Henni Setia Ningsih, Masatsugu Oishi, Yu-Jen Chou

**Affiliations:** a Department of Mechanical Engineering, National Taiwan University of Science and Technology No. 43, Sec. 4, Keelung Road Taipei 10607 Taiwan yu-jen.chou@mail.ntust.edu.tw +886-2-2737-6492; b Graduate School of Technology, Industrial and Social Sciences, Tokushima University Josanjima-cho Tokushima 770-8506 Japan

## Abstract

Due to the bioactive properties that stimulate the growth of new bones, bioactive glasses (BGs) have been widely used in fields of medical science. Yet, the lack of mechanical properties limits the application of BGs. Therefore in this study, thin film 58S BGs were prepared *via* magnetron sputtering with customed targets from spray-dried powders. The preparation method of radio-frequency magnetron sputtering was used with the advantages of excellent uniformity, high adherence, and precise thickness control over other deposition methods. The crystallographic information, thin film morphology, and elemental composition of the as-deposited thin film BGs were characterized *via* X-ray diffraction, scanning electron microscopy, and energy dispersive spectroscopy, respectively. Furthermore, the *in vitro* bioactivity was examined using simulated body fluid according to Kokubo's protocol, whereas the *in vitro* cell viability was assessed following the 3-(4,5-dimethylthiazol-2-yl)-2,5-diphenyltetrazolium bromide assay. Finally, the results showed the successful preparation of thin film 58S BG specimens with bioactivity and non-toxicity, and their corresponding formation mechanisms and property correlations were discussed.

## Abstract 2

Thin film bioactive glasses were prepared *via* magnetron sputtering through a custom spray dried powders. Due to the bioactive properties, the materials have demonstrated their potential for bone implant applications.

## Graphical abstract

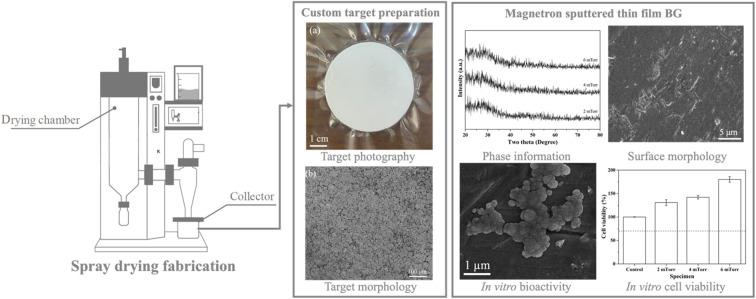

## Introduction

Since Hench's original report in 1969, bioactive glasses (BGs) have attracted tremendous attention and have shown their potential as promising materials with their superior properties such as bioactivity, biocompatibility, and biodegradability.^1^ Therefore in the past few decades, there have been extensive developments in the fields of biomedical engineering,^2,3^ with practical applications including bone implants, dental fillers, and drug carriers.^4–6^ To date, most research on BGs has primarily focused on the conventional melt-quenching method, with secondary attention given to the sol–gel preparation.^7^ Emerging alternative glass preparation technologies, such as spray pyrolysis,^8,9^ or spray drying,^10,11^ are also noteworthy. These methods produce BG specimens in bulk or powder form. BGs, whether in bulk, powder, or scaffold form-often fabricated by freeze drying^12^ and various 3D printing techniques^13^ have demonstrated positive *in vitro* and *in vivo* feedback, though their applications are currently limited to low-load bearing scenarios.^14,15^ In contrast, the functionalization of BG coatings onto metal implants has been considered as an alternative approach to obtain rapid integration with living tissue, which could help bone regeneration and offer support for bone growth.^16^ Hence, there has been enormous attention given to the study of bioactive coatings onto metal implants, while various deposition methods have demonstrated promising biological results.^17,18^

To obtain metallic implants with good osteointegration, various thin film deposition methods, such as pulsed laser deposition,^19^ pulse electron deposition,^20^ magnetron sputtering,^21^*etc.*, have been used for the preparation of thin film BGs. Among these methods, plasma spraying is the most widely used and the only method that has received approval from the US Food and Drug Administration (FDA).^22^ It offers numerous advantages, including a wide range of substrates that can be coated with various coatings (polymers, metals, and ceramics) and cost-effective surface treatment for a variety of applications.^23^ However, it has a number of drawbacks, such as poor adhesion between the substrate and coating, phase change caused by exposure to high temperature, deviations in the coating density, poor microstructural control, and microcracks.^24^ Additionally, studies have employed pulsed laser deposited to fabricate thin film BGs onto Ti alloys,^25,26^ while Bellucci *et al.* demonstrated the preparation of 45S5 by pulse electron deposition method showing its high efficiency and optimized stoichiometry conservation.^20^ However, the high laser power might lead to micro-cracks and internal pores as well as damage the substrate, whereas the pulse electron source may not be widely available. In brief, although each deposition method has its own pros and cons, all these researches indicated the importance of BG coating for implant application development.

Apart from the deposition methods mentioned, magnetron sputtering is a potential technique that has been applied in fields of thin film technology owing to its versatility in automation, great uniformity and adherence, and precise control of specimen thicknesses.^27^ For instance, Stan *et al.* reported a series of optimization with their deposition system and demonstrated the prospect of adjusting the structure, morphology, and bioactivity of BG films.^28,29^ Moreover, Popa *et al.* functionalized dental screws with BG and showed positive effects on the adhesion and proliferation of human dental pulp stem cells.^30^ To date, the preparation of thin film BGs using the magnetron sputtering technique has employed only melt-quenched BG materials as cathode targets. The use of sol–gel, spray pyrolysis, or spray drying BGs for the thin film fabrication by magnetron sputtering remains unexplored. Exploring the sputtering deposition mechanisms when employing these latter types of BGs, along with the resulting structural, compositional, and biological features, could be significantly important for the future development of implant coatings.

Therefore, in the present work, we aimed to prepare thin film 58S BGs *via* magnetron sputtering based on a single target formulation. Among the various BG compositions, 58S stands out as particularly advantageous due to its low tendency to crystallize and its ability to maintain a high rate of HA formation, leading to enhanced cell mineralization.^31^ By fabricating the BG powders with the spray drying method, which is considered the most efficient method for converting the suspension into a free-flowing powder, the resulting powders were cold pressed into green compacts and sintered into custom targets. Then, sputtering of thin film 58S BGs was conducted, and the crystallographic information, surface morphology, and elemental composition were examined *via* X-ray diffraction (XRD), scanning electron microscopy (SEM), and energy dispersive spectroscopy (EDS), respectively. For the evaluation of *in vitro* bioactivity tests, both SEM and EDS were used to observe the formation of hydroxyapatite, while the *in vitro* cell viability tests were assessed following a 3-(4,5-dimethylthiazol-2-yl)-2,5-diphenyltetrazolium bromide (MTT) assay. Finally, formation mechanisms and their related properties of all specimens were discussed.

## Experimental details

### Synthesis

First, 58S BG powder (SiO_2_ : CaO : P_2_O_5_ = 60 : 35 : 5 in mol%) was fabricated using the spray drying method.^10^ Initially, a precursor solution was prepared by adding 238.1 g of tetraethyl orthosilicate (Si(OC_2_H_5_)_4_, 99.9%, Showa, Japan), as the source of SiO_2_, into 400.0 g of ethanol (C_2_H_5_OH, 99.5%, Echo, Taipei, Taiwan), and followed by adding 157.4 g of calcium nitrate tetrahydrate (Ca(NO_3_)_2_·4H_2_O, 98.5%, Showa, Japan) and 34.7 g of triethyl phosphate ((C_2_H_5_)_3_PO_4_, 99.0%, Alfa Aesar, UK) as the source of CaO and P_2_O_5_. Note that all precursors were obtained in analytical grade. Additionally, 0.5 M hydrochloric acid (HCl, 37.0%, Honeywell, United States) was used to adjust the pH of the solution to 2.5 for the optimization of precursor dissolution. Finally, by mixing the precursor solution with de-ionized water till 1000 mL, the final solution was stirred for a day to achieve homogeneity. For the spray drying method, the prepared precursor solution was atomized *via* a rotary disc atomizer operated at 20 000 rpm into fine droplets. The droplets were then directed into the spray drying equipment (SDD0-03, IDTA Machinery Co. Ltd, Taiwan) *via* a peristaltic pump with a flow rate of 20 mL min^−1^. Then, the inlet and outlet temperatures were set at 200 and 80 °C for the spray drying chamber, while the spray-dried powder was collected using an attached cyclone collector. Next, for the preparation of the target, the spray-dried BG powder was filtered with 140 mesh to acquire uniform particle sizes. Then, a 2.15-inch cylinder mold was filled with 17 g of BG powder and cold pressed with 150 MPa to obtain the green compact with a thickness of 3 mm. At last, heat treatment of 900 °C was carried out for impurity calcination and target sintering.

For the deposition of thin film 58S BG specimens, pure Ti plates (Circle & Cycle Ltd, Taiwan) of 10 × 10 mm^2^ were used as substrates (Polished, with surface roughness of around 150 nm). All substrates were cleaned *via* ultrasonic for 5 min in ethanol, then fixed onto a stainless-steel holder in the chamber, with a target–substrate distance of 10 cm, and dried in a flow of nitrogen. During the sputtering process, the vacuum chamber was evacuated to 1 × 10^−2^ mTorr (0.0013 Pa) using a diffusion pump. The target was pre-sputtered for 5 min before each deposition to eliminate possible surface impurities. For all thin film BG specimens, the RF power (L200A01FM, MeiVac Inc, USA) and Ar gas flow were set at 100 W and 30 sccm (standard cubic centimeters per minute), with three working pressures of 2, 4, and 6 mTorr (0.266, 0.533, and 0.799 Pa), and the duration of deposition was kept at 12 h.

### Characterizations

First, characterizations of crystallographic information were conducted with XRD (D8 Discover, Bruker, Germany) for the custom target and all as-deposited thin film BG specimens. The instrument is equipped with a Cu-Kα source with Ni-filter, giving a wavelength of 1.54 Å. Note that for these characterizations only, thin film BG specimens were sputtered onto (100) Si substrates (Semiconductor Wafer Inc., Taiwan) in order to remove the strong diffraction of the pure Ti substrate. The diffraction patterns were acquired with an incident angle of 0.5°, while the collection angle was ranging from 10 to 80° with step size set at 0.05° and a collection time of 2.5 s per step.

Next, the surface morphology was examined by SEM (FEI Quanta 3D FEG, FEI, United States). Initially, SEM micrographs were captured *via* a field-emission probe for the observation of surface morphology. In addition, cross-sectional specimens were prepared for the measurements of specimen thicknesses. In addition, EDS spectra were obtained for the analysis of elemental compositions. For the statistical measurements of average thickness and elemental compositions, the measurements were carried out by sampling more than three regions across the whole specimen to ensure its reliability. Further, Fourier transform infrared spectroscopy (FTIR) was performed using a SHIMADZU Tracer-100 spectrometer in reflection mode with an Attenuated Total Reflectance (ATR) suite equipped with a Ge crystal prism to identify the functional groups in the thin film BG specimens. The analysis was conducted with a resolution of 4 cm^−1^ across a range of 4000 to 600 cm^−1^.

Evaluation of *in vitro* bioactivity of all thin film BG specimens were conducted based on Kokubo's protocol.^32^ By immersing the specimen into 10 mL of simulated body fluid (SBF) following the formula *v*_SBF_ = 100 mm *S*_a_, which has ion concentrations similar to human blood plasma (as shown in Table 1), the specimen was held at 37 °C for 14 d in a thermostatic orbital shaker (S300R, Firstek Scientific, Taiwan). Meanwhile, it is worth mentioning that the SBF solution was refreshed every 24 h to simulate our body condition. The resulting specimen was gathered and cleaned three times using both acetone and de-ionized water to clean any remaining salts, then were placed into an oven set at 70 °C to dry for 24 h. Lastly, for the observation of bioactivity, SEM and EDS were employed for direct observation and the Ca/P ratio of the formation of HA.

**Table 1:** Ionic concentration of SBF and human blood plasma

Ion	Simulated body fluid	Human blood plasma
Na^+^	142.0	142.0
K^+^	5.0	5.0
Mg^2+^	1.5	1.5
Ca^2+^	2.5	2.5
Cl^−^	147.8	103.0
HCO_3_^−^	4.2	27.0
HPO_4_^2−^	1.0	1.0
SO_4_^2−^	0.5	0.5
		Unit: mmol L^−1^

Nano-indentation tests were performed using a nano-indenter (TI-980 TriboIndenter, Hysitron Inc., United States). During indentation, a diamond Berkovich indenter with a 5-5-5 loading function was utilized. A loading/unloading rate was set to 200 μN s^−1^, reaching a maximum load of 1000 μN to evaluate the hardness and elastic modulus of each sample. It's important to ensure that the indentation depth remains below 10% of the specimen's thickness to avoid the influence from the substrate.

Finally, *in vitro* cell viability was assessed *via* MTT assay according to the ISO protocol 10993-5.^33^ To start with, osteoblast cells (MC3T3-E1, Taipei Medical University) were cultivated in a minimum essential medium (MEM) and were then transferred into several 24-well plates at a density of 2 × 10^4^ cells per well. In the meantime, thin film BG specimens were sterilized in an autoclave at 130 °C and soaked in MEM for 24 h to form the test solutions. The solutions were incubated with cells at 37 °C for 72 h, the media were then removed, and followed by adding the MTT reagents. Then, all plates were transferred into a CO_2_ incubator set at 37 °C and incubated for another 2 h, while formazan crystals were formed by the healthy osteoblast cells. Finally, by adding dimethyl sulfoxide to dissolve the formazan crystals, the optical densities of all test solutions were measured *via* a microplate spectrophotometer (Cytation 3, Bio TEK, USA) at a wavelength of 550 nm.

### Results

**Fig. 1:**
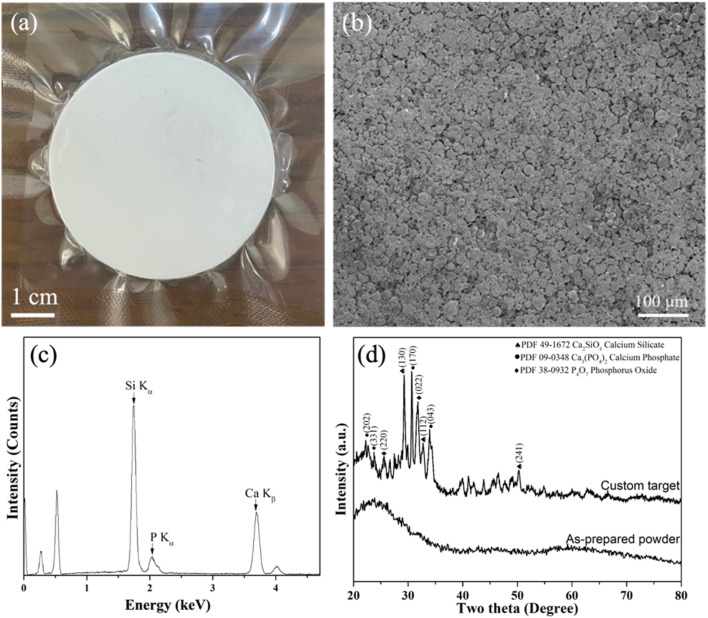
Information on the custom target from spray dried BG powders. (a) photograph, (b) SEM image, (c) EDS spectrum, and (d) XRD pattern.

To start with, Fig. 1 shows the information on the prepared custom target. Firstly, the photograph of the custom target is shown in Fig. 1(a), indicating a successful preparation of a diameter of 2-inch. Additionally, the SEM image and EDS spectrum are shown in Fig. 1(b) and (c), showing the surface morphology of the sintered spray-dried BG powders with the corresponding Si-Kα, P-Kα, and Ca-Kα peaks observed at 1.74, 2.06, and 3.69 keV, respectively. Further, elemental compositions were computed and shown in Table 2, indicating the ratio of Si : Ca: P is 56.01 ± 0.82 : 36.39 ± 2.22 : 7.60 ± 1.51, which shows a similar ratio as compared to the nominal 58S (Si : Ca: P = 57.14 : 33.33 : 9.52). Lastly, the XRD patterns of the as-prepared powder and the custom target are shown in Fig. 1(d). The results showed that the as-prepared powder exhibited an amorphous structure with a broad band around 20 to 40°, which was reported in our previous study.^34^ In contrast, after the heat treatment of 900 °C, multiple phases of calcium silicate (JCPDF 49-1672), calcium phosphate (JCPDF 09-0348), and phosphorus oxide (JCPDF 38-0932) could be found. In addition, the crystallinity of the custom target was computed as 28.0%.

**Table 2:** Chemical compositions of the target and as-deposited thin film BG specimens

Specimen	Si	Ca	P
Nominal	57.14	33.33	9.52
Target	56.01 ± 0.82	36.39 ± 2.22	7.60 ± 1.51
2 mTorr	59.03 ± 0.38	33.98 ± 0.23	6.99 ± 0.62
4 mTorr	59.43 ± 0.36	32.98 ± 0.54	7.59 ± 0.38
6 mTorr	58.78 ± 0.56	33.55 ± 0.23	7.67 ± 0.77
			Unit: at%

**Fig. 2:**
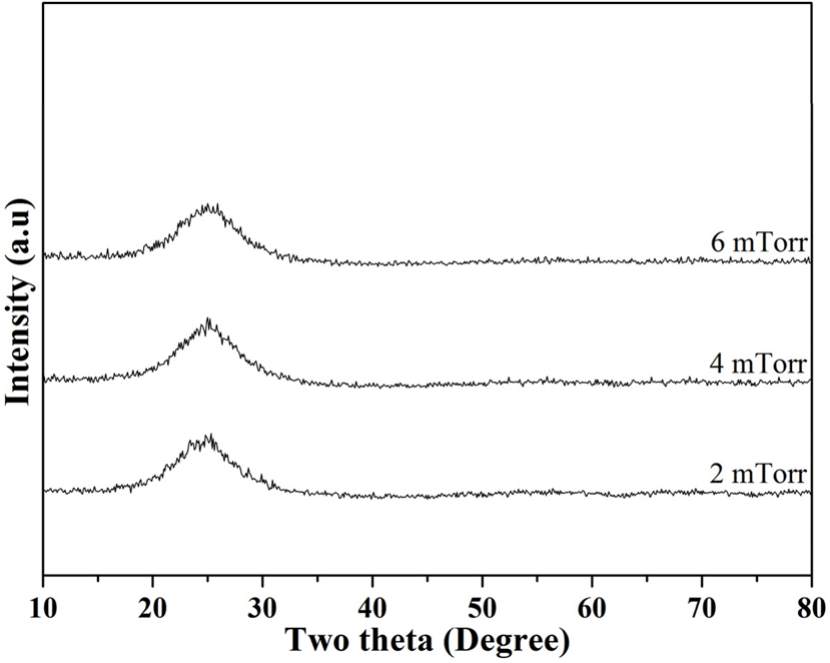
XRD patterns of as-deposited thin film 58S BG specimens with working pressures of 2, 4, and 6 mTorr.

Next, Fig. 2 shows the XRD patterns of the as-deposited thin film BG specimens with working pressures of 2, 4, and 6 mTorr (0.266, 0.533, and 0.799 Pa). Initially, it could be seen from the graph that there were no distinct diffraction intensities could be observed from angles of 10° to 80° for the thin film BG specimen sputtered with 2 mTorr (0.266 Pa). In contrast, a broad hump could be observed between angles of 20° to 30°, indicating the amorphous structure. In addition, as the working pressure increased, thin film BG specimens sputtered with 4 and 6 mTorr (0.533, and 0.799 Pa) showed similar results with no distinct diffractions. In brief, the XRD results suggested that all thin film BG specimens were successfully deposited with amorphous structures.

**Fig. 3:**
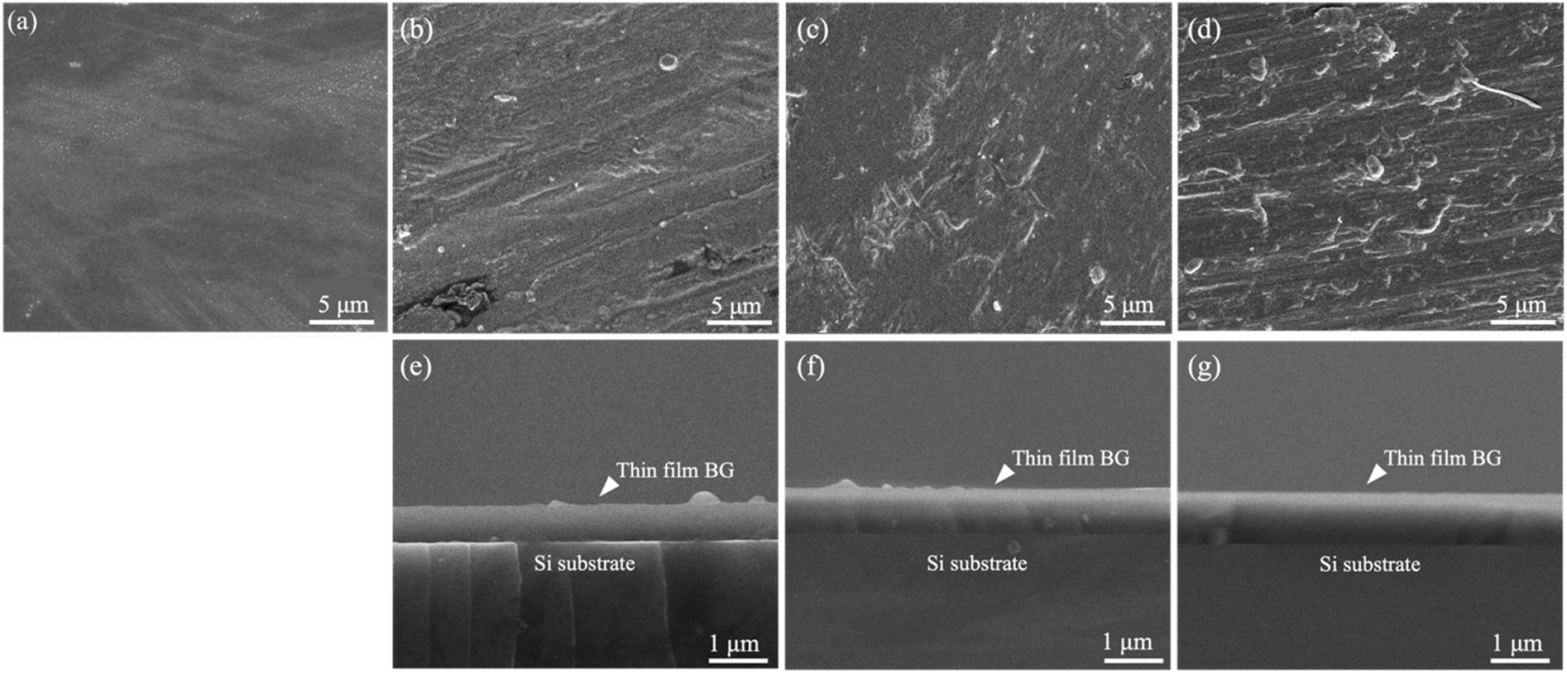
SEM images of (a) undeposited Ti substrate and the top and cross-sectional view of as-deposited thin film BG specimens with working pressures of (b) and (e) 2 mTorr, (c) and (f) 4 mTorr, and (d) and (g) 6 mTorr.

Further, Fig. 3 shows the SEM images of all thin film BG specimens. Initially, Fig. 3(a) showed the undeposited Ti substrate without any in-growths or precipitation. Next, for the thin film BG specimen sputtered with 2mTorr (0.266 Pa), a smooth surface morphology could be observed in Fig. 3(b), indicating a uniform growth of BG. In addition, with the increased working pressure, some spherical in-growths on the surface could be found (Fig. 3(c) and (d)). The measured in-growths diameters vary between 0.4 to 2.1 μm, showing a rise as the working pressure increases. Furthermore, thicknesses of thin film BG specimens were measured *via* the cross-sectional images as shown in Fig. 3(e)–(g). By sampling five regions across the whole specimen, the averaged film thicknesses were statistically measured, which resulted in 651 ± 29, 807 ± 17, and 906 ± 15 nm for the thin film BG specimens deposited with 2, 4, and 6 mTorr (0.266, 0.533, and 0.799 Pa), respectively.

**Fig. 4:**
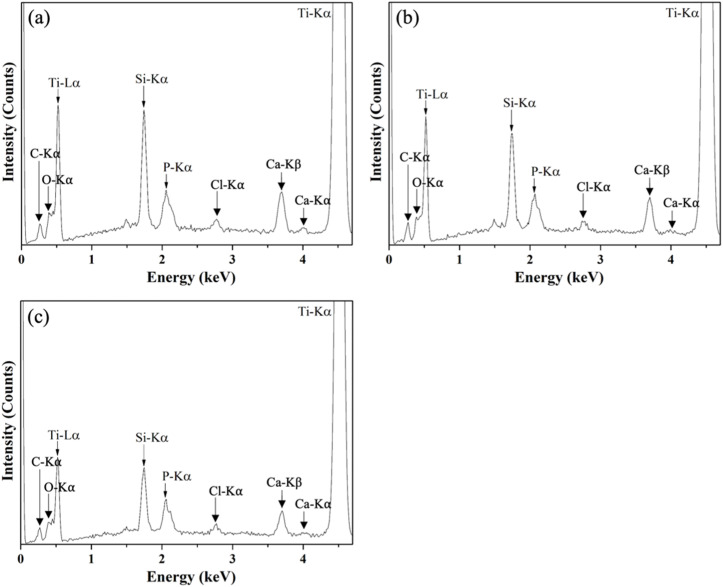
EDS spectra of as-deposited thin film BG specimens with working pressures of (a) 2 mTorr, (b) 4 mTorr, and (c) 6 mTorr.

The elemental compositions of the target and as-deposited thin film BG specimens were characterized *via* EDS, and the recorded spectra are shown in Fig. 4. The results showed that the peaks of C-Kα, O-Kα, Ti-Lα, Si-Kα, P-Kα, Cl-Kα, Ca-Kβ, Ca-Kα, and Ti-Kα could be found at 0.28, 0.55, 0.51, 1.74, 2.03, 2.65, 3.69, 4.01, and 4.51 keV, respectively, from all thin film BG specimens. Further, elemental compositions were computed and shown in Table 2. It could be seen from the table that the composition of the target showed a similar ratio as compared to the nominal 58S composition (Si : Ca: P = 57.14 : 33.33 : 9.52). Then, for the thin film BG specimens, the Si : Ca: P ratios are 59.03 ± 0.38 : 33.98 ± 0.23 : 6.99 ± 0.62, 59.43 ± 0.36 : 32.98 ± 0.54 : 7.59 ± 0.38, and 58.78 ± 0.56 : 33.55 ± 0.23 : 7.67 ± 0.77 for the thin film BG specimens with working pressures of 2, 4, and 6 mTorr (0.266, 0.533, and 0.799 Pa), respectively. Increased concentrations of around 2 to 4% were observed on both Si and Ca, whereas the concentrations of P were decreased by around 5% among all thin film BG specimens. In brief, it could be observed that the composition of all thin film BG specimens was stoichiometrically transferred to the specimens with around 5% variance.

Additionally, the hardnesses and elastic modulus of all specimens were determined using nano-indentation. To start with, by sampling five areas from each specimen, the average hardness of the pure Ti substrate was measured as 2.01 ± 0.40 GPa. Further, significant increases in specimen hardnesses could be observed from the thin film BG specimens, showing 4.68 ± 0.57, 4.98 ± 0.26, and 4.55 ± 0.10 GPa for the thin film BG specimens prepared at working pressures of 2, 4, and 6 mTorr (0.266, 0.533, and 0.799 Pa), respectively. Further, the elastic moduli were measured as 68.4 ± 0.4, 67.6 ± 0.6, and 69.6 ± 0.4 GPa for the thin film BG specimens prepared at working pressures of 2, 4, and 6 mTorr (0.266, 0.533, and 0.799 Pa), respectively.

**Fig. 5:**
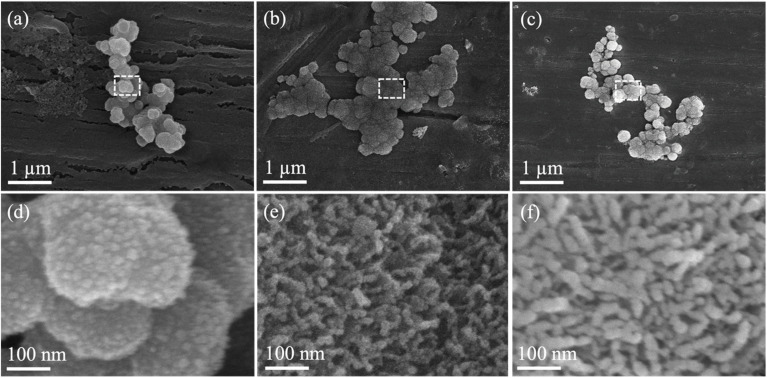
SEM images of thin film BG specimens with working pressures of (a) 2 mTorr, (b) 4 mTorr, and (c) 6 mTorr after SBF immersion for 14 d. (d)–(f) Show magnified images of the insets in micrographs (a)–(c), respectively.

**Fig. 6:**
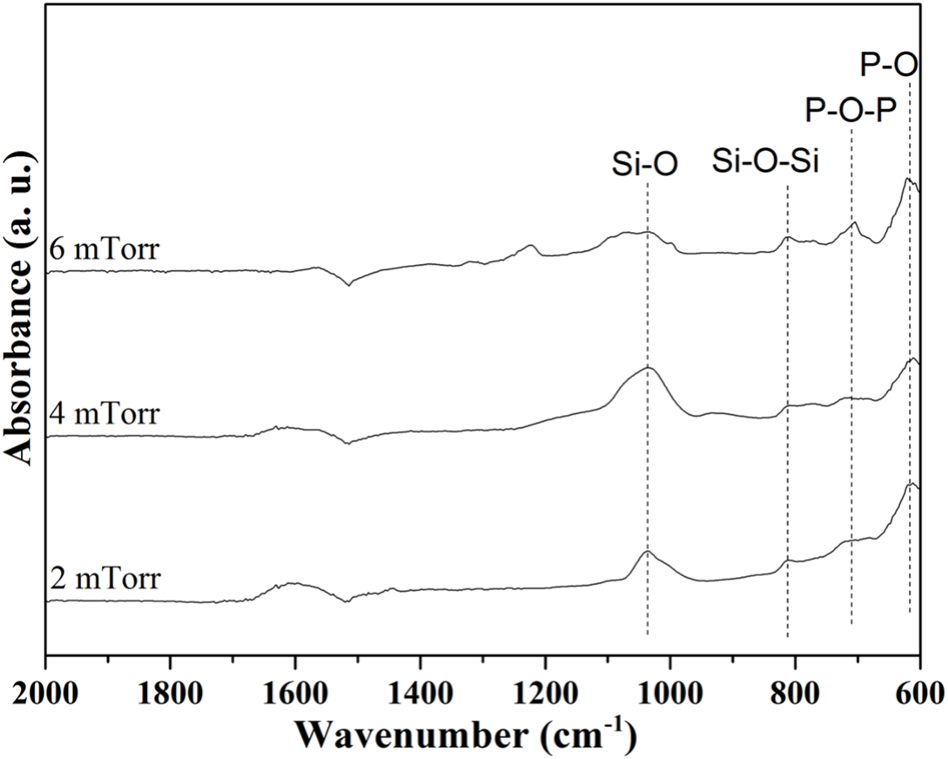
FTIR spectra of hin film BG specimens with working pressures of 2 mTorr, 4 mTorr, and 6 mTorr after SBF immersion for 14 d.

For the *in vitro* bioactivity, evaluations were conducted after SBF immersion for 14 d, and the resulting SEM images are shown in Fig. 5, along with their magnified images of the marked area. Initially, it could be observed that the formation of needle-shaped hydroxyapatite deposits on the surface of all the thin film BG specimens. In contrast to the as-deposited thin film BG specimens with working pressures of 4 and 6 mTorr (0.533, and 0.799 Pa), the hydroxyapatite deposits had increased, as compared to the as-deposited thin film BG specimens with working pressures of 2 mTorr (0.266 Pa). These results evidenced the interaction between the surface of the thin film BG specimens and the SBF solution, culminating in hydroxyapatite formation. Meanwhile, the results from EDS analysis showed that the Ca/P ratios for the thin film BG specimens with working pressures of 2, 4, and 6 mTorr (0.266, 0.533, and 0.799 Pa) are 1.37, 1.52, and 1.45, respectively. In addition, FTIR spectra were employed to examine the chemical bonds of the formation of HA, and the results are shown in Fig. 6. To start with, the main bands related to the silica network could be observed at around 1050 cm^−1^ (corresponding to Si–O stretching). While peaks around 810 cm^−1^ were found, which are corresponding to the symmetric stretching vibrations of Si–O–Si groups. Further, the peaks around 610 cm^−1^ are characteristic of P–O bending of PO_4_^3−^ groups, which correspond to the calcium phosphate-based compounds. Meanwhile, a supplementary band around 700 to 750 cm^−1^ was observed, which could be attributed to bands involving ν4 bending of orthophosphates and/or pyrophosphates. In summary, the calcium phosphate compound deposits on the surface of all thin film BG specimens were observed after 14 d of SBF immersion, indicating potential bioactivity.

**Fig. 7:**
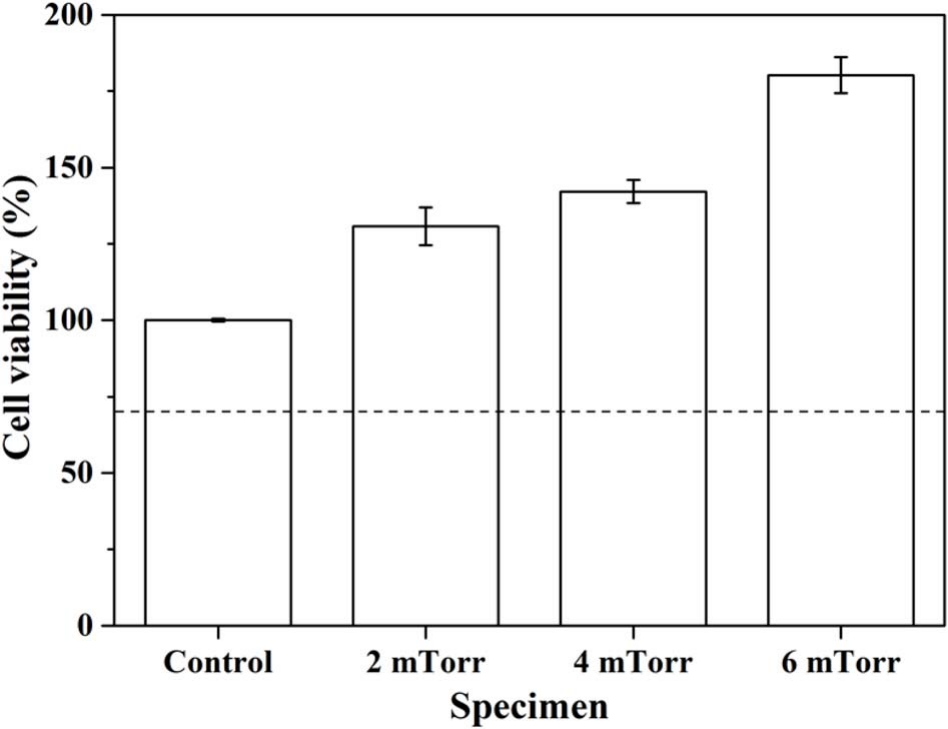
Cell viability of as-deposited thin film BG specimens with various working pressures.

Finally, Fig. 7 shows the *in vitro* cell viability results of all thin film BG specimens. Based on the ISO protocol 10993-5, the cell viability was computed against the control sample as a percentage, while the computed value must pass the level of 70% (marked in the dashed line) in order to be regarded as a non-toxic material. As shown in Fig. 7, the results revealed that all percentages of cell viabilities were above 70%, satisfying the ISO standard requirement. In addition, the cell viabilities of thin film BG specimens deposited with 2, 4, and 6 mTorr (0.266, 0.533, and 0.799 Pa) are 130.8 ± 6.2, 142.2 ± 3.8, and 180.2 ± 5.9%, respectively, showed higher viability as compared to the control sample (100%), and could be considered as an evident proliferation effect. In brief, the *in vitro* cell viability results indicated that all thin film BG specimens exhibit non-toxicity and could help the proliferation of osteoblast cells.

### Discussion

First, we discuss the formation details of the custom target. To start with, it is worth noting that a target shrinkage of around 7–8% was measured after target sintering. Thus, a custom mold of 2.15-inch was used to counter the shrinkage. Additionally, based on the SEM images shown in Fig. 1(b), the target porosity was calculated by ImageJ software as 28.1 ± 2.5%. This might not be ideal and could be further improved by employing other processes, such as cold isostatic pressing (CIP) and hot isostatic pressing (HIP). Additionally, other approaches of using finer powders could enhance packing density, while applying sintering aids could promote better densification. Regarding the crystallization, Hadem *et al.* reported that the crystallization temperature of the 58S BG was measured as 825 °C.^35^ Meanwhile, the sintering temperature of 900 °C used in this work is higher than the crystallization temperature, thus resulting in the formation of various calcium and phosphorus phases.

Next, for the thin film BG specimens, the surface morphology was discussed. Based on the SEM images of the as-deposited thin film BG specimens as shown in Fig. 2, specimens that sputtered with higher working pressure resulted in a rough surface with increased agglomeration found on its surface. The formation of these aggregates could be described as the results of the surface energy minimization process which was reported by Stan *et al.*^28^ Owing to the short mean free path of the sputtered atoms under the higher working pressure, which have higher tendency to collide with other atoms in the chamber, the atoms lost their energy during the collision and could not be deposited onto the substrate effectively. In addition, the transverse diffusion ability of the atoms is decreased, resulting in the formation mechanism of island growth, therefore leading to rough surface morphology. Regarding the EDS results, apart from the primary elements of Ti, Si, Ca, and P, a Cl-Kα intensity at 2.65 keV was observed in the spectra of all films. This could be attributed to the residual chlorine remaining from the hydrochloric acid used to adjust the pH of the precursor solution.

Next, for the thin film thicknesses, the deposition rates in this study were computed as 54.2, 67.2, and 75.5 nm h^−1^, showing slower rates as compared to previous studies.^36,37^ This is owing to the long target–substrate distance (10 cm) in the current sputtering system. Following Meng *et al.*,^38^ when the mean free path of the sputtering atoms is smaller than the target–substrate distance, the energy of the atoms is not sufficient for an effective deposition, which led to low deposition rate and low film density. Additionally, with the increasing working pressure, increased specimen thicknesses could be obtained, this was reported by Stuart *et al.*,^39^ demonstrated that the number of backscattered atoms would increase at a higher rate than the number of ejected atoms as the working pressure increased, thus leading to an increase in deposition rate. Meanwhile, one can observe the positive effect of thin film BG deposition on hardness. The results showed an increased hardness of up to 5 GPa as compared to pure Ti of 2 GPa, showing a similar trend with Popa *et al.*^40^ In addition, a study by Teghil *et al.* showed that 45S5^®^ Bioglass coatings by pulsed laser deposition (PLD) possess hardnesses of around 2 to 4 GPa,^41^ Kawagichi *et al.* investigated the surface modification through electrophoretic deposition (EPD) of 45S5® Bioglass coatings, demonstrating hardnesses of 0.5 to 2.0 GPa and elastic moduli between 70 and 130 GPa, using either direct current (DC) or alternating current (AC),^42^ and Tang *et al.* prepared 6P57 BG coatings *via* spray deposition and examined their hardnesses and elastic moduli under different load levels (50, 200, 500 μN), showing hardnesses ranging from 3.4 to 7.8 GPa and elastic moduli from 80 to 90 GPa.^43^ These reports are comparable to the current study, indicating its readiness for biomedical applications. Moreover, Miki-Yoshida and Aandrade^44^ reported that the deposition rate decreases as the film thickness increases, suggesting that the deposition rate dependent on the film thickness. The mechanism that explain the thickness-dependent growth rate is similar to that of inhibited particle growth.^45^ The growth rate should be thickness-dependent, as the crystallite faces necessitate repeated nucleation events in order to progress to the subsequent stages of growth.

Finally, we discussed the *in vitro* bioactivity and the *in vitro* cell viability of the thin film BG specimens. Initially, the SEM results showed significant differences in the surface morphology after SBF immersion. The as-prepared thin film BG specimen has a smoothed surface with some distribution of small-sized in-growths. In contrast, the specimen after SBF immersion showed a dense layer with uniformly distributed precipitations, such surface morphology is commonly observed as the Ca-deficient apatite. Additionally, the EDS analysis showed a significant difference in Ca/P ratio as compared to as-deposited thin film BG (from 4.34–4.86 to 1.37–1.52). Although the Ca/P ratios fell short of the nominal Ca/P ratio of 1.67 for hydroxyapatite, possibly due to the effect of the BG thin films underneath, the results in this study demonstrated the preferential development of the apatite layer following an extended immersion in SBF.

## Conclusions

In this study, thin film BG specimens were successfully prepared *via* magnetron sputtering technique. The resulting crystallographic composition, thin film morphologies, specimen thicknesses, atomic compositions, and hardness were characterized by XRD, SEM, EDS, and nano-indentation. The results demonstrated the successful preparation of custom targets from spray drying powders and its ability to adjust the morphology, composition, and thickness of thin film BG specimens *via* altering the working pressure, and the formation mechanism was discussed. Finally, for the *in vitro* bioactivity and cell viability, the SEM, EDS, and MTT results confirmed the non-toxic and potential bioactivity of all thin film BG specimens after 14 d of SBF immersion. With advantages such as chemical flexibility, controllable morphology, and rapid kinetic process of the spray drying technique over melt-quench and sol–gel methods, these findings reveal the potential development of thin film BG coatings and could be considered as a possible candidate for bone implant applications.

## Data availability

All data underlying the results are available as part of the article and no additional source data are required.

## Conflicts of interest

On behalf of all authors, the corresponding author states that there is no conflict of interest.
